# Predominant Asymmetrical Stem Cell Fate Outcome Limits the Rate of Niche Succession in Human Colonic Crypts

**DOI:** 10.1016/j.ebiom.2018.04.017

**Published:** 2018-04-25

**Authors:** Craig Stamp, Anze Zupanic, Ashwin Sachdeva, Elizabeth A. Stoll, Daryl P. Shanley, John C. Mathers, Thomas B.L. Kirkwood, Rakesh Heer, Benjamin D. Simons, Doug M. Turnbull, Laura C. Greaves

**Affiliations:** a LLHW Centre for Ageing and Vitality, Newcastle University Institute for Ageing, The Medical School, Newcastle upon Tyne NE2 4HH, UK; b Wellcome Centre for Mitochondrial Research, Institute of Neuroscience, Newcastle University, Newcastle upon Tyne NE2 4HH, UK; c Swiss Federal Institute of Aquatic Science and Technology, Department of Environmental Toxicology, Dübendorf, Switzerland; d Northern Institute for Cancer Research, Newcastle University, Newcastle upon Tyne NE2 4AD, UK; e Institute of Neuroscience, Newcastle University, Newcastle upon Tyne NE2 4HH, UK; f Institute of Cell and Molecular Biosciences, Newcastle University Institute for Ageing, Campus for Ageing and Vitality, Newcastle University, Newcastle upon Tyne NE4 5PL, UK; g Human Nutrition Research Centre, Institute of Cellular Medicine, Newcastle University, Newcastle upon Tyne NE4 5PL, UK; h Cavendish Laboratory, Department of Physics, University of Cambridge, J.J. Thomson Avenue, Cambridge CB3 0HE, UK; i Wellcome Trust/Cancer Research UK Gurdon Institute, University of Cambridge, Tennis Court Road, Cambridge CB2 1QN, UK; j Wellcome Trust/Medical Research Council SC Institute, Cambridge CB2 1QR, UK

**Keywords:** Intestine, Stem cell, Cell division, Mitochondria, Stem cell fate

## Abstract

Stem cell (SC) dynamics within the human colorectal crypt SC niche remain poorly understood, with previous studies proposing divergent hypotheses on the predominant mode of SC self-renewal and the rate of SC replacement. Here we use age-related mitochondrial oxidative phosphorylation (OXPHOS) defects to trace clonal lineages within human colorectal crypts across the adult life-course. By resolving the frequency and size distribution of OXPHOS-deficient clones, quantitative analysis shows that, in common with mouse, long-term maintenance of the colonic epithelial crypt relies on stochastic SC loss and replacement mediated by competition for limited niche access. We find that the colonic crypt is maintained by ~5 effective SCs. However, with a SC loss/replacement rate estimated to be slower than once per year, our results indicate that the vast majority of individual SC divisions result in asymmetric fate outcome. These findings provide a quantitative platform to detect and study deviations from human colorectal crypt SC niche homeostasis during the process of colorectal carcinogenesis.

## Highlights

• Asymmetric stem cell fate outcome predominates in human colonic crypts.
• Human colonic epithelium harbours ~5–7 effective stem cells per crypt.
• Low stem cell replacement rates limit niche succession.

Stamp et al. report the use of genetic clonal lineage tracing in humans, using mitochondrial enzyme activity as a clonal mark, and show that there are 5–7 effective stem cells within the colorectal crypt stem cell niche and that predominant asymmetrical stem cell fate with a low stem cell replacement rate can explain niche succession. Understanding the basic biological processes of colonic crypt stem cell homeostasis provides a platform to investigate and potentially modulate the deviations from such processes which occur during colorectal carcinogenesis.

## 1 Introduction

The human colorectal epithelium contains approximately 10 million crypts which, in common with mice, are thought to be maintained by pools of adult stem cells (SCs) supported by a niche environment at the crypt base [19]. Resolving the mechanisms responsible for the maintenance of the human colorectal crypt SC niche is vital, not only as a model of human SC biology, but also as a basis for investigating deviations from homeostasis that are known to occur during the initiation of colorectal carcinogenesis [1,31].

In the mouse, previous studies have used a genetic labeling system based on a ubiquitous promoter to trace the long-term behavior of intestinal crypt SCs and their differentiating progeny. These studies have proposed that the maintenance of the crypt relies on a process of “neutral cell competition” in which pools of equipotent SCs at the crypt base compete for limited niche access [17]. In this model, SC division is perfectly compensated by the differentiation and loss of proximate cells so that the overall size of the crypt SC population remains constant over time, the condition of homeostasis. At the clonal level, stochastic SC loss and replacement leads to the chance expansion and contraction of SC clones until the clone is altogether lost through differentiation or the crypt becomes fully monoclonal (fixed), a process known as “niche succession”. These early findings were corroborated by parallel genetic labeling studies in mouse using a multicolored confetti reporter construct under the control of the Lgr5 promoter, which addressed both clonal and mosaic density labelling [17,27]. Although these lineage tracing investigations could resolve the existence and basis for neutral clone dynamics, and the multipotency of intestinal crypt SCs, the range of the crypt SC compartment remained uncertain. However, based on the range of expression of the putative SC marker, Lgr5, researchers placed a figure of around 16 SCs in the mouse small intestinal crypt [17,27].

Subsequent tracing studies, based on the continuous sporadic activation of a reporter allele through a frameshift mutation, suggested that the maintenance of the colonic and small intestinal crypt may, in fact, rely on as few as 5–7 “effective” SCs [13], questioning the function of the wider Lgr5-expressing crypt-base population. In the same study, the loss/replacement rate of this effective crypt SC compartment was found to be around ~0.3 per day in mouse colon [13]. With a SC division rate of around once per day, this suggests that a significant fraction of SC divisions result in symmetric fate outcome. Finally, in the most recent chapter of the debate, the combination of intravital imaging of the mouse small intestine with genetic labelling using an Lgr5 promoter enabled the detailed reconstruction of individual lineage and positional information in time-lapse over a 3–4 day time course [24]. This ground-breaking study showed that Lgr5+ cells positioned towards the base of the crypt experience a short-term bias towards renewal, while those positioned near the border are, by location, primed for displacement from the niche, differentiation and loss. However, through cell rearrangements at the crypt base, SCs can exchange position and, as a result, reassign reversibly their fate bias. Within this framework, it was shown that a dynamically heterogeneous SC population, extending across the range of Lgr5+ cells (and perhaps beyond), functions long-term as a smaller effective equipotent SC population with a dynamics described by the original neutral drift model [24]. In other words, the one-dimensional neutral drift model with its 5–7 effective crypt SCs is a caricature of a more complex dynamics that captures quantitatively the long-term dynamics of crypt SC clones.

Although these studies have provided important insights into the functional behaviour of SCs during maintenance in the mouse intestine, such transgenic lineage tracing approaches are clearly not feasible in humans. Moreover, although the sporadic acquisition of somatic nuclear DNA point mutations confers a hereditary mark that could provide access to lineage information at the clonal level, the low mutation rate combined with the resolution of current sequencing technologies makes its application challenging in large cohorts. Alternative approaches to study SC lineages in human intestine have used methylation patterns as surrogate markers. Intriguingly, these studies hint at a remarkably slow rate of niche succession in the human colonic crypt, with typical time-scales estimated to be as long as 8.2 years [12,33]. However, the rapid turnover of methylation marks questions the reliability of this approach [9].

Age-related somatic mitochondrial DNA (mtDNA) mutations are more frequent than mutations of the nuclear genome. These mutations cause a defect in oxidative phosphorylation (OXPHOS) which is readily observed using dual-color cytochrome *c* oxidase (COX)/succinate dehydrogenase (SDH) histochemistry [29], providing an alternative clonal labeling strategy. In human colon, the abundance of OXPHOS-deficient crypts increases with age, with the first clones detectable at ~20 years of age [10,11,29]. Both partially and fully OXPHOS-deficient crypts are detected suggesting that, initially, OXPHOS-deficient SCs occupy only part of the colonic crypt but, through a process of loss and replacement, may go on to colonize the entire crypt. Crucially, mtDNA sequencing of OXPHOS-deficient cells taken from partially and fully deficient crypts has confirmed that OXPHOS-deficient cells within a crypt are clonally derived and the under lying mtDNA mutations are somatic in origin with different crypts containing separate mtDNA mutations. [1,29] These observations suggest that mitochondrial OXPHOS-deficiency may be a reliable tool for mapping human colonic crypt SC fate and dynamics, as the observation of both partially and fully deficient crypts and their relative frequency can inform on the multiplicity of effective SC number and the dynamics of niche succession in human colonic crypt.

Based on this approach, a contemporary study of aged human colon used 3D reconstructions of partially OXPHOS-deficient crypts to map the size and shape of clonal imprints on the crypt wall [1]. Based on the neutral drift model of crypt SC dynamics, it was argued that the spatial profile of the clonal patch provided a historical record of the size and activity of labelled SCs at the crypt base. Quantitative analysis of the variation of the angular clonal patch width as a function of position along the axis of the crypt was shown to be consistent with neutral drift dynamics of the resident self-renewing population, with estimated effective crypt SC loss/replacement rates comparable to that reported in mouse, and more than an order of magnitude faster than those estimated from methylation studies [1].

To resolve these seemingly contradictory findings from previous investigations, here, using OXPHOS-deficiency as a crypt SC lineage marker, we have studied changes in the abundance and size distribution of clonal patches in sections of human colon across the entire adult life-course to determine the number and replacement rate of normal human colorectal crypt SCs. Our detailed quantitative studies corroborate the early estimate of a very slow rate of effective SC loss/replacement. We discuss the implications of these findings for the pattern of intestinal SC fate behaviour.

## 2 Materials and Methods

### 2.1 Study Participants

Colorectal mucosal biopsies were collected from the same anatomical site (10 cm from the anal verge) from participants (n = 148, age range 17–78 years) undergoing colonoscopy for disturbed bowel function in whom no evidence of bowel disease was identified (BORICC 1 Study). Subjects were divided into age groups as follows; 17–20 years (n = 2), 21–30 years (n = 10), 31–40 years (n = 25), 41–50 years (n = 44), 51–60 years (n = 37), 61–70 years (n = 21), 71–80 years (n = 9) Ethical approval was obtained from the Northumbria NHS Trust Local Research Ethics Committee (Project reference NLREC2/2001). Informed consent was obtained from all subject recruited to the study.

### 2.2 Cyctochrome *c* Oxidase/Succinate Dehydrogenase (COX/SDH) Histochemistry

Colon samples were mounted for sectioning and frozen in isopentane previously cooled to −190 °C in liquid nitrogen. Cryostat sections (12 μm) were cut onto glass slides and incubated in COX medium (100 μM cytochrome *c*, 4 mM diaminobenzidine tetrahydrochloride and 20 μg.ml^−1^ catalase in 0.2 M phosphate buffer pH 7.0) at 37°C for 50 min. Sections were washed in phosphate buffered saline, pH 7.4 (3 × 5 min) and incubated in SDH medium (130 mM sodium succinate, 200 μM phenazine methosulphate, 1 mM sodium azide, 1.5 mM nitroblue tetrazolium in 0.2 M phosphate buffer pH 7.0) at 37°C for 45 min. Finally, sections were washed in phosphate buffered saline, pH 7.4 (3 × 5 min), dehydrated in a graded ethanol series (70%, 95%, 2 × 100%), cleared in Histoclear® (National Diagnostics, USA) and mounted in DPX. Sections were imaged and the proportion of OXPHOS deficiency in each crypt quantified using StereoInvestigator software.

### 2.3 Modelling Niche Succession Within Human Colonic Crypts

We developed a multi-scale stochastic model of mtDNA mutation clonal expansion by random genetic drift and SC niche succession within colonic crypts. The model was designed and run in MATLAB (version 7.14.0.739 MathWorks, Massachusetts, United States) and was based solely on experimentally derived parameters. The model source code is available from the authors upon request. Full details are provided in the supplementary materials.

## 3 Results

### 3.1 Analysis of OXPHOS Deficiency in Human Colorectal Epithelium from Healthy Adults

**Fig. 1:**
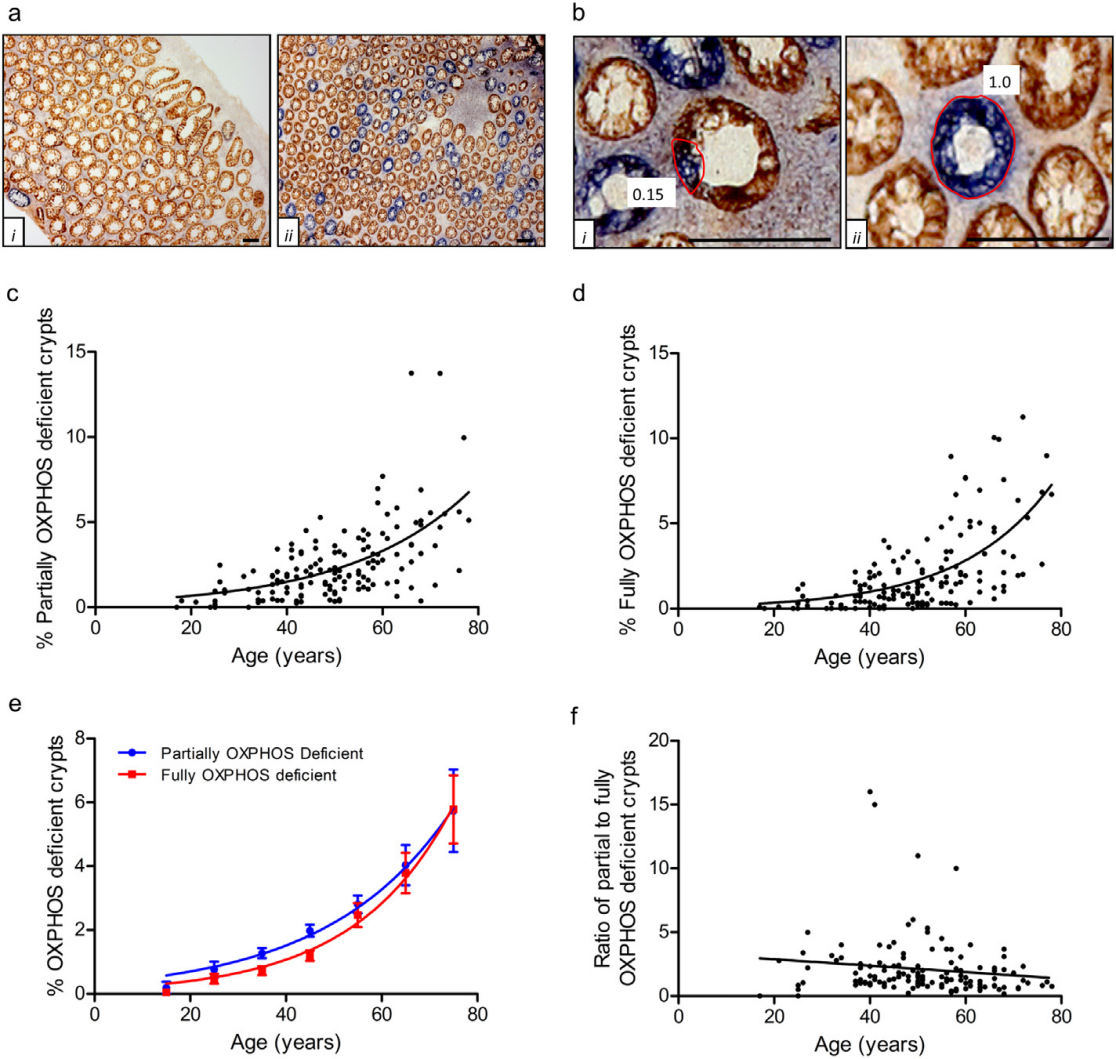
Analysis of OXPHOS-deficient clone size in an ageing series of human samples. **a**: Low magnification images giving an overview of COX deficiency observed in a subject aged **i**) 25 and **ii**) 70. OXPHOS deficient clones are blue, OXPHOS normal clones are brown. **b**: (**i**) Partially OXPHOS deficient crypt with an OXPHOS deficiency fraction estimated to be 0.15. Note that, in line with the model of neutral drift, the labelled cells form a contiguous patch around the circumference of the crypt. (**ii**) Fully OXPHOS-deficient crypt, OXPHOS deficiency fraction = 1.0. Percentage of (**c**) partially OXPHOS deficient crypts and (**d**) fully OXPHOS deficient crypts observed within colon biopsies in patients of different ages (n = 148). Both plots fit best to an exponential growth curve (R^2^ = 0.3595 and R^2^ = 0.4000 respectively). (**e**): Frequency of partially and Fully OXPHOS deficient crypts grouped into 10 year age brackets (17–20 years (n = 2), 21–30 years (n = 10), 31–40 years (n = 25), 41–50 years (n = 44), 51–60 years (n = 37), 61–70 years (n = 21), 71–80 years (n = 9)) and plotted against the percentage of OXPHOS deficient crypts. The data fit best to an exponential growth curve (R^2^ = 0.9912 for partial crypts and R^2^ = 0.9917 for fully deficient crypts. (**f**) The ratio of partially to fully OXPHOS deficient crypts does not change significantly with age (linear regression analysis, p = .1066).

Long-term lineage tracing analysis using COX/SDH histochemistry was performed on 148 histologically normal colonic biopsy samples from participants aged 17–78 years showing no evidence of colorectal pathology. For each biopsy section, the proportion of blue COX-deficient/SDH-positive labelling [29] was measured in individual crypts (Fig. 1a and b). Over 150,000 crypts were analysed in total. In the case of clusters of adjacent crypts bearing fully OXPHOS-deficient cells, the whole cluster was counted as a single clone as the vast majority of these events have been shown previously to have the same clonal origin, being the product of naturally occurring crypt fission events [10]. For simplicity, we do not take into account the intriguing possibility of crypt fusion noting that, based on estimates in mouse, the relative abundance of such events is likely to be small [5].

When combined across patient samples, our results showed an age-related increase in the average number of both partially and fully OXPHOS-deficient crypts (Fig. 1c, R^2^ = 0.360 and Fig. 1d, R^2^ = 0.400, respectively). Due to inherent patient-to-patient variability in the frequency of mtDNA mutation, data were grouped by decade revealing an exponential-like growth characteristic in the abundance of OXPHOS-deficient crypts (Fig. 1e, R^2^ = 0.991 for partial crypts, R^2^ = 0.992 for fully deficient crypts). Surprisingly, in contrast to parallel studies based on the sporadic activation of a frameshift gDNA mutation [13], we found no significant change in the ratio of partial to fully OXPHOS-deficient crypts with age (Fig. 1f, linear regression analysis, p = 0.1066), a point to which we return below.

Due to the time required for mtDNA mutations to accumulate above the threshold level at which OXPHOS-deficiency can be detected, as well as our previous mtDNA sequencing analyses, we reasoned that the overwhelming majority of fully and partially labelled crypts must be rooted in individual clonal events associated with the SC compartment. Therefore, in line with previous analyses [1,13], we proposed that a section through the circumference of the crypt would translate to a “snap shot” of the activity of the crypt SC compartment earlier in time, from which the relative proportion of OXPHOS-deficient SCs per crypt at that time could be inferred.

**Fig. 2:**
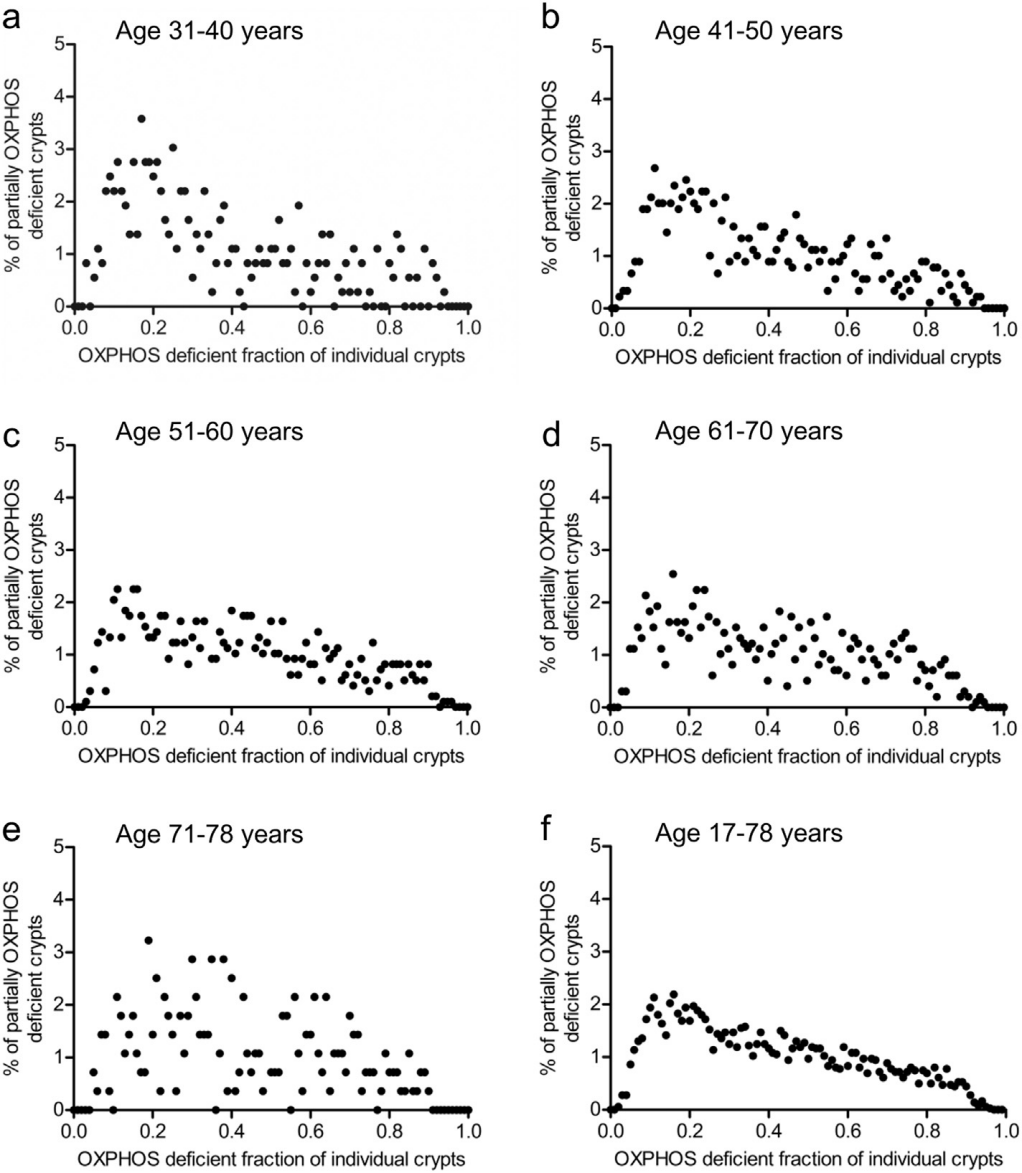
**Frequency distribution of the fraction of OXPHOS deficiency in all partial crypts.** Data are grouped by age (a–e) then plotted as the whole data set (f). The <30 age bracket had too few data points for age-group analysis, but was included in the total data analysis.

Analysis of partially OXPHOS-deficient crypts revealed a broad distribution of labelling around the crypt circumference with a pronounced peak visible at an OXPHOS-deficiency fraction of ~0.15–0.2, decaying monotonically and near-linearly with increasing size, and with a profile that was independent of age (Fig. 2a–f). Equating the position of the peak to the typical clonal output of a single OXPHOS-deficient SC within a crypt, this observation suggested a crypt composition involving some 5–7 effective SCs (viz. 1/0.2 to 1/0.15) within each human colonic crypt, a figure broadly consistent with that reported for mouse colon [13].

### 3.2 The Dynamics of OXPHOS-deficient Clones Is Consistent With a Slow Rate of Crypt SC Loss and Replacement in the Human Colorectal Epithelial Crypt

To understand how these findings can be used to define the dynamics of the colonic crypt SC pool, we turned to a quantitative statistical analysis of the clonal data based on the canonical model of neutral crypt SC dynamics in mouse [17]. In this framework, the effective crypt SC population is modelled as a one-dimension annulus of cells that wrap around the circumference of the crypt base. During the course of epithelial turnover, SC division may result in asymmetric fate outcome in which one daughter remains within the effective crypt SC pool, while the other is displaced from the “niche” and enters into a differentiation program, dividing and differentiating into the migration streams of functional cells that progress along the walls of the colonic crypt. Alternatively, SC division may result in displacement and loss of a neighbouring SC from the pool – the process of neutral cell competition.

To address the viability of this neutral drift model in the human colon, we considered its extension to the current mtDNA labelling system. In contrast to the genetic labelling system in mouse, the emergence of OXPHOS-deficient clones involves a two-stage process in which marked cells have to first amplify the abundance of mutant mtDNA molecules above the threshold of detection, followed by the individual clone dynamics of mutant cells. Before considering the full dynamics of these sequential processes, we first considered a minimal extension of the neutral drift model in which the emergence of OXPHOS-deficient cells above detection threshold was modelled as an abrupt and irreversible stochastic event, conditioned by the observed nonlinear accumulation rate of mtDNA mutations in our biological analyses (Fig. 1e) (Supplementary Theory Note 1). Within the framework of the neutral drift model, the clonal fixation of an OXPHOS-deficient crypt is an irreversible event. Therefore, from the observed increase in the fraction of fixed crypts (Fig. 1e), we could infer the rate of increase in the effective time-dependent clonal induction rate, averaged across the patient samples. Based on the experimental findings, we estimated an induction rate of OXPHOS-deficient SCs that, during adulthood, increases empirically as *R*_0_*e*^*ηt*^, where *η* = 0.05 per year and *R*_0_/*N* = 0.01 per crypt per year, where *N* is the effective crypt SC number (see Supplementary Theory Note 1 for details). With an effective SC number of *N* *=* *5*, this translates to an induction rate of OXPHOS-deficient SCs of 0.14 per crypt per year at age 20 rising to around 1.7 per crypt per year at age 70. (Note that, over time, in the paradigm of neutral SC competition, only 1/N of these marked cells will give rise to clones that become fixed within individual colonic crypts.)

We then considered how the abundance and size distribution of partially labelled crypts could provide insight into clonal dynamics. Within the framework of the neutral drift model [17], the clonal dynamics are specified by just two parameters, the number of effective SCs around the crypt circumference, *N*, and their rate of loss and replacement by neighbours, *λ*; a rate that is bound by the cell division rate but, through asymmetrical division, may be much lower. To accommodate the potential for fate bias induced by OXPHOS-deficiency, we further allowed for the possibility that mutant SCs replace neighbouring non-mutated SCs with a higher efficiency than a non-mutated neighbour, providing an additional fit parameter.

Based on this model, we then determined the predicted distribution of partial crypt sizes as a function of age. For a time-independent clonal induction rate and strictly neutral dynamics, the model predicts that the distribution of partial crypts should decay linearly with size, independent of the SC loss/replacement rate, taking the maximum value at a fraction *1/N* of the crypt circumference and reaching its minimum value at *1–1/N* (Supplementary Theory Note 1), a behaviour highly consistent with the measured distribution (Fig. 2). Indeed, analysis of the partial clone size distribution suggested only a weak departure from the predicted linear size dependence (Supplementary Theory Note 1). Notably, our analysis showed that even a small variation in fate bias of OXPHOS-deficient cells translated to a large change in the shape of the size distribution of clones suggesting that, if present at all, any bias of OXPHOS-deficient clones must be small (Supplementary Theory Note 1 and Fig. S1), establishing OXPHOS-deficiency in the colon as an approximately neutral mark.

Finally, from the theoretical analysis of the neutral drift model subject to the observed exponentially increasing rate of OXPHOS-deficient cell accumulation (Supplementary Theory Note 1), we noted that the abundance of partial crypts in tissue depended sensitively on the effective crypt SC loss/replacement rate. Analysis of the model showed that the observed increase and relative frequency of fully and partially labelled crypts could be accurately predicted by the neutral drift dynamics model with an effective SC loss/replacement rate of around 0.6 per year (Supplementary Theory Note 1), almost two orders of magnitude smaller than reports of the typical proliferative rate of crypt base progenitors in human colon [21].

### 3.3 Multi-Scale Stochastic Modelling of mtDNA Clonal Expansion and SC Dynamics

**Fig. 3:**
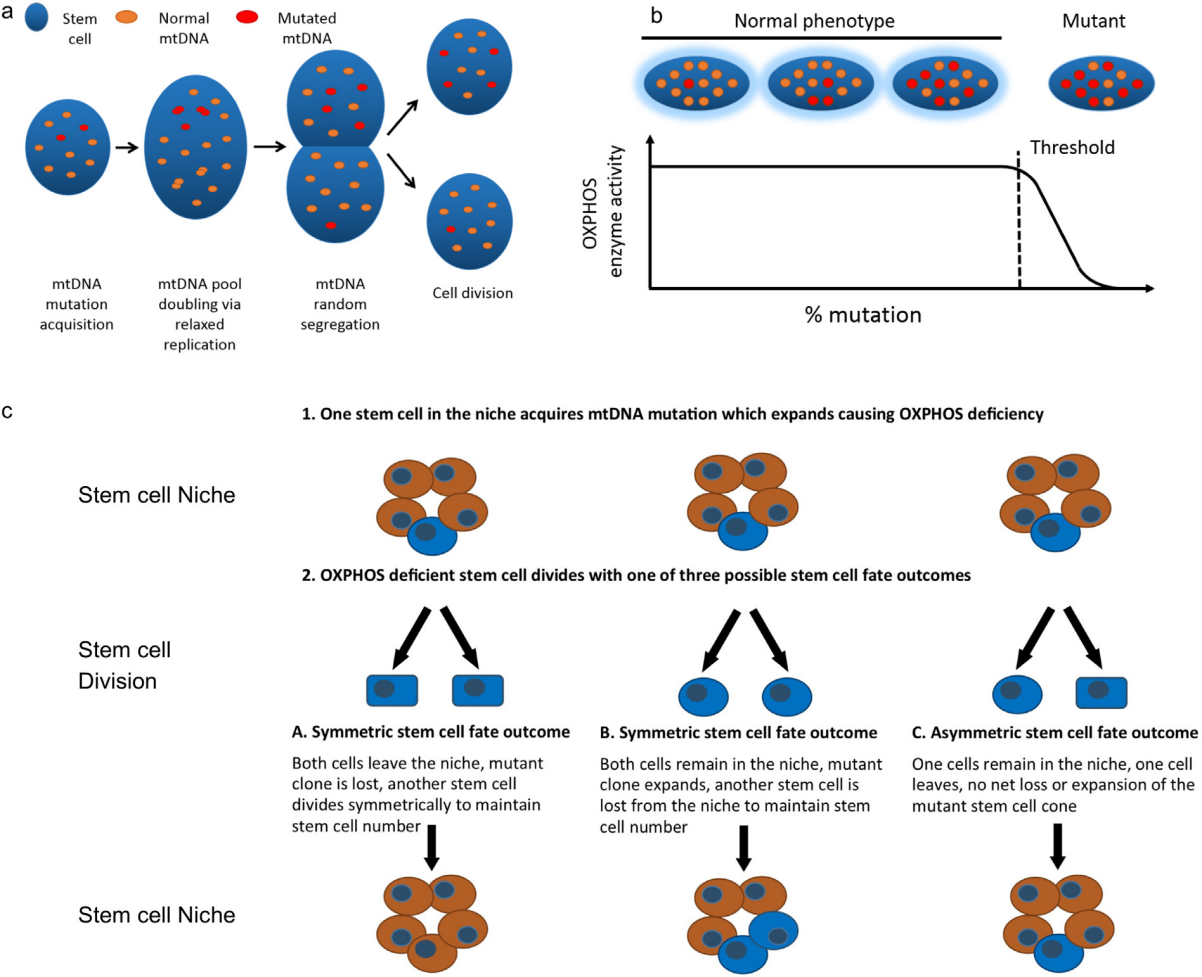
Modelling neutral drift stem cell dynamics of OXPHOS deficient stem cells within human colonic epithelium. (**a**) In the model, in each stem cell (SC) mtDNA mutations are randomly generated according to a defined mutation rate, then mutated mtDNA molecules can clonally expand within dividing SCs via relaxed replication and random segregation upon cell division. (**b**) When the fraction of mutated mtDNA reaches a threshold in a SC, the cell become OXPHOS-deficient. (**c**) In the model, in each round of cell division, all SCs divide and then half are lost from the niche to retain the original number of SCs. SC fate outcome can either be symmetric or asymmetric. Symmetric SC fate outcome division can result in SC clone contraction as well as expansion, whereas asymmetric SC fate outcome only results in the retention of the resultant SC clone.

Although these findings point at a process of neutral dynamics of the effective SC compartment with a slow SC loss/replacement rate, our quantitative analysis assumed a clean separation between the internal dynamics of mtDNA mutation accumulation within individual cells and subsequent clonal dynamics of OXPHOS-deficient cells. To integrate these components and assess the validity of this approximation, we employed a more refined multi-scale one-dimensional stochastic model simultaneously simulating *i*) the clonal expansion of mtDNA mutations within individual crypt SCs to the level at which OXPHOS-deficiency is observed and *ii*) neutral SC dynamics within the colonic crypt SC niche. The three levels of the model are depicted in Fig. 3 and are detailed in Supplementary Theory Note 2. All parameters in the model were based on published experimental data (Table 1). Where the data from the literature gave multiple estimates, e.g. for mtDNA mutation rate, and mtDNA copy number per cell, a parameter scan was performed to obtain the best fit to the experimental data.

**Table 1:** Simulation parameters used in the multi-scale stochastic model of neutral drift stem cell dynamics.

Parameter	Value	Semantic	Reference
Stem cell division time	1/week	Time between consecutive stem cell divisions.	[21]
mtDNA copy number	200	Number of mtDNA molecules contained within each stem cell of the crypt.	[2,6,32]
Mutation rate	1.0 × 10^−5^ (starting rate at time = 0) increasing exponentially to 6.0 × 10^−5^ (time point = 80 years)/mtDNA/replication	The probability that a mtDNA replication event results in a mtDNA molecule becoming mutated.	[6,7,26]
OXPHOS-deficiency threshold	75%	The percentage threshold of mutated mtDNA molecules that produces an OXPHOS-deficient stem cell.	[15,25]
Simulation runs	30,000	The number of simulation runs (crypts) required for data convergence.	
Number of divisions	4171	The number of stem cell divisions equal to the lifetime of an 80 year old crypt. Based on the stem cell division time.	
Stem cell number	5	The number of stem cells simulated per crypt.	
Asymmetric cell fate outcome probability	0–1	The probability that when a stem cell divides there is an asymmetrical stem cell fate outcome, whereby one cell remains in the niche and one cell leaves the niche	
Symmetric cell fate outcome probability	0–1	The probability that a when stem cell divides there is a symmetric stem cell fate outcome, whereby either both cells remain in the niche or both cells are instantly expunged from the stem cell niche.	

**Fig. 4:**
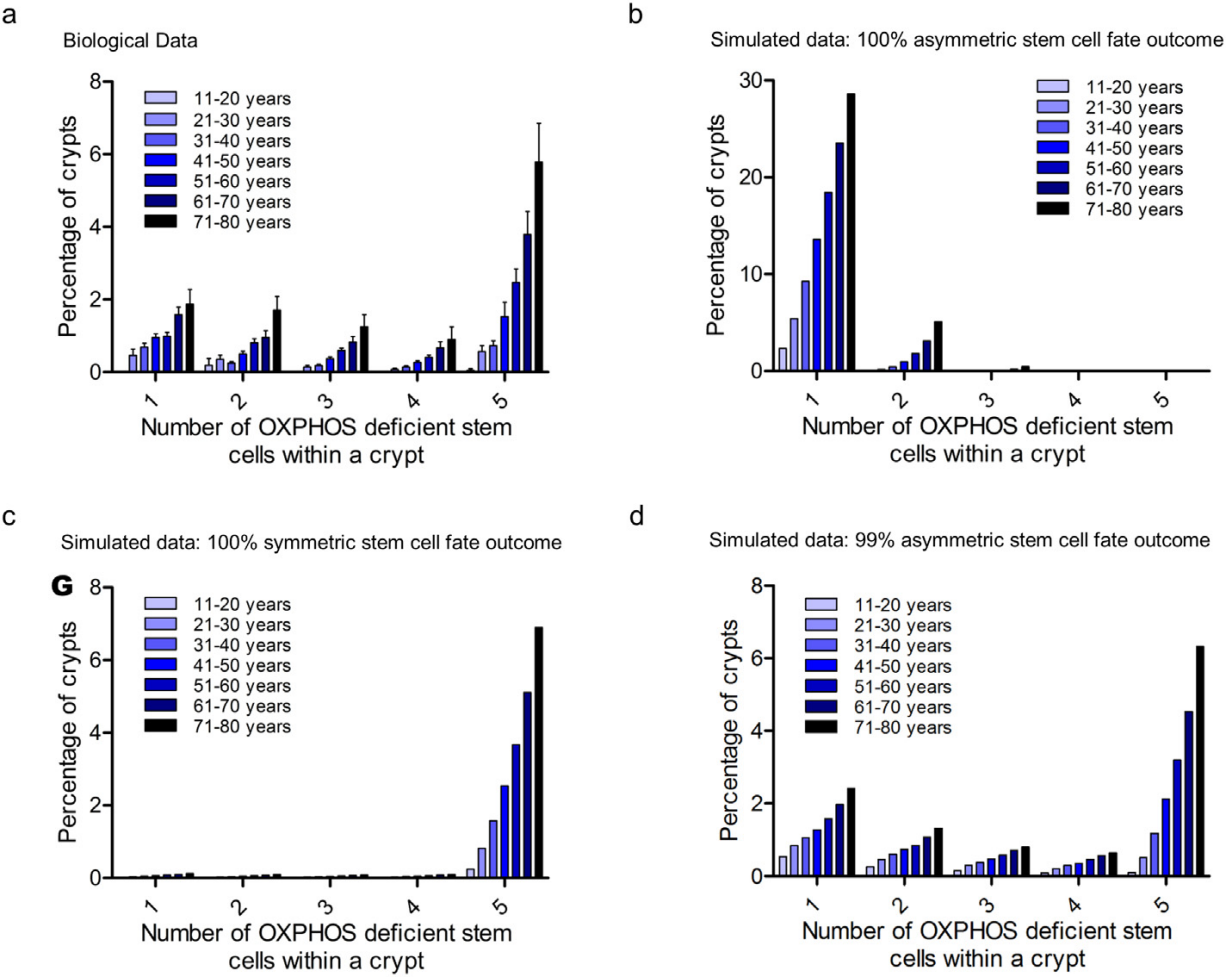
Experimental data compared with results of a stochastic neutral drift model of stem cell dynamics and OXPHOS deficiency. (**a**) Experimental OXPHOS-deficiency data grouped by decade of age binned into increments of 0.2 of fraction of OXPHOS deficiency and converted to number of deficient stem cells (SCs), in this case 5. There is an exponential increase in the percentage crypts with 1, 2, 3, 4 and 5 deficient SCs (R^2^ = 0.923, 0.967, 0.987, 0.983, 0.993 respectively). Modelling results with (**b**) 100% asymmetric SC fate outcome, (**c**) 100% symmetric SC fate outcome and (**d**) best fit of 99% asymmetric SC fate outcome corresponding to a SC replacement rate of 0.52 per year.

The model was initially based on there being *N* *=* *5* SCs in the crypt SC niche (Fig. 2) and each crypt was followed for >4000 rounds of SC division, equivalent to ~1 round of SC division per week up to an age of ~80 years. MtDNA mutation rate was estimated following parameter scans of published rates to be 1.0 × 10^−5^ at time zero increasing to 6.0 × 10^−5^ at 80 years, and mtDNA copy number was set at 200 per crypt SC [2,6,32]. The mutation threshold for OXPHOS deficiency was set to 75% mutant mtDNA molecules per cell [15,25]. As the effective number of crypt SCs is a critical factor in the model, a second model was developed to determine the probability that the proportion of OXHOS-deficiency observed above the level of the SC compartment was representative of the SC number. This model took into account the possibility of lateral dispersion of the OXPHOS-deficient SC progeny as the cells migrate up the crypt (Fig. S2). When models were generated for 3–8 SCs and compared to the frequency distribution of the biological data shown in Figs. 2, 5 SCs gave the best fit to the data (Fig. S2). The human OXPHOS data were then grouped by decade, binned into increments of 0.2 and then converted to number of SCs; subsequent models were compared with these data (Fig. 4a).

Results of the model simulations firstly confirmed that, as expected, in a model based exclusively on invariant asymmetric SC fate outcome, the rate of acquisition of mutations was insufficient to explain the accumulation of OXPHOS-deficient crypts (Fig. 4b). Fully OXPHOS-deficient crypts were never generated. Only crypts with one OXPHOS-deficient SC, and occasional crypts with two OXPHOS-deficient SCs arising from independent mtDNA mutations were observed. These data are entirely consistent with our previous sequencing analyses where fully OXPHOS-deficient crypts were shown to be clonal, therefore experimentally ruling out invariant asymmetry as the predominant mode of SC division [11,29]. In a model involving only symmetric SC division leading to stochastic loss/replacement of neighbours, very few partially OXPHOS-deficient crypts were observed due to rapid niche succession times (Fig. 4c). Finally, when fitting the model with the percentage of asymmetric SC fate outcome as a free parameter to the measured OXPHOS-deficiency, the alignment to the observed human in situ data was achieved with a SC fate outcome of 99% asymmetry and 1% SC loss/replacement, with 5 effective crypt SCs in the niche (Fig. 4d). This corresponded to a SC replacement rate of 0.52/year, consistent with the analytical results obtained using the simplified modelling scheme above.

## 4 Discussion

We have used mtDNA mutations that result in OXPHOS-deficiency as a lineage tracing strategy to investigate long-term SC dynamics in the human colorectal crypt. Using a large cohort of human colorectal mucosal biopsies with representation across the whole adult life-span, we have provided in vivo evidence that the human colorectal crypt is characterized by approximately 5 effective SCs. Combining the relative abundance and size distribution of partially mutated crypts, our quantitative analysis shows that the long-term maintenance of the crypt epithelium relies on a similar, but far slower, pattern of neutral drift dynamics to that resolved in mouse [13,17,24,27]. With an effective SC loss/replacement rate of around once per 1–2 years, the process of niche succession – the clonal fixation of an individual crypt by the progeny of a single SC – typically takes many years to complete. The sensitivity of the clone size distribution, as well as the relative rate of accumulation of partial and fully clonal crypts, to potential fate biases in OXPHOS-deficient cells provides the means to establish, *post-hoc*, the neutrality of the clonal mark; a useful consistency check given the concerns about neutrality of OXPHOS-deficiency. Future advances in DNA sequencing technology will allow the results here to be checked against clonal data obtained from the quantitative study of the allele fractions of individual genomic DNA mutations obtained from individual crypts.

If the rate of effective colorectal crypt SC division mirrors that of their differentiating progeny, estimated by BrdU incorporation at around once per 5–7 days [20], the current study suggests that, in contrast to mouse, the vast majority (~99%) of effective SC divisions result in asymmetric fate outcome, leaving the clonal configuration of the SC niche unchanged. This result suggests species-specific differences in the balance of effective SC fate. How could such a high proportion of asymmetric SC divisions be regulated? In some developmental contexts, such as the *Drosophila* neuroblast, the spontaneous asymmetric segregation of fate determinants promote asymmetric fate outcome [23]. In other contexts, such as the *Drosophila* testis, the predominance of asymmetric fate outcome is enforced through physical interactions with the niche environment – somatic hub cells – that promote mitotic spindle-orientation during cell division [16], leaving one daughter remote from the niche and thereby primed for differentiation. In the present context, it may be that terminally differentiated crypt base secretory cells that contribute to niche function present long-lived physical barriers that serve to orient SC division at the crypt base along the axis of the crypt, leaving one of the daughter cells positioned away from the crypt base and poised for differentiation while the other remains at the base, biased for renewal [22,24].

Alternatively, the turnover of the human colon might rely on the reversible transfer of SCs between activity and quiescence, explaining the strikingly low average rate of effective SC replacement. Although human BrdU incorporation studies indicate that the majority of progenitors at the crypt base are actively cycling [21], the existence of a low fraction of slow-cycling reserve SCs has been identified in the mouse [4,8,30]. The reliance of a maintenance mechanism based on the activity of a near-quiescent SC population would provide a strategy to protect the integrity of the long-lived self-renewing compartment from the acquisition of deleterious mutations. Further biological studies are required to confirm our theoretical analyses as well as elucidate the biological mechanisms underlying the human colonic crypt stem cell fate bias predicted by our investigations. For example, attempts to identify human colonic crypt SCs and careful quantification of the spindle pole orientation during SC division should be made as well as analyses of the spatial distribution of daughter cells following SC division.

Although our findings are in broad agreement with the estimate of typical niche succession times based on the study of methylation patterns [33], our findings seem to contradict previous analyses based on the 3D reconstructions of the “wiggle” patterns of OXPHOS-deficient clones used to estimate directly the rate of SC loss/replacement [1]. We have previously carried out similar 3D reconstructions and can confirm that the “wiggles” detected by Barker et al. were also present in our studies [29]. One possibility to explain the differences between the current analyses and those of Barker et al. is that the observed large-scale and seemingly rapid variations in the circumferential size of clones reflect bursts of proliferative activity of the transit-amplifying SC progeny as they emerge from the niche domain at the crypt base. Alternatively, the progeny of the SC compartment may not conform to a “classic” phase of transit-amplification, but instead may display a high degree of self-renewal potential, i.e. as effective SC progeny enter this compartment, they compete neutrally with progenitor neighbours with a high rate of loss/replacement, leading to the observed wiggle-like variation of the clonal imprints on the walls of the crypt. Indeed, such behaviour would mirror the recently described organization of the stomach corpus [14]. Whether the same kind of dynamics persists in the human small intestine remains an interesting question for future studies.

Initiation of colorectal carcinogenesis is thought to occur via accumulation of genetic aberrations primarily in the intestinal SCs that confer a selective clonal advantage [3,18]. Quantification of SC replacement rates in mouse models with common initiating mutations in *APC* and *Kras* showed an increased probability of, and a lower time to, clonal fixation [28,31]. We have shown OXPHOS-deficiency to be a reliable marker of SC fate outcome in human tissue with the observed clone size distribution evidencing the neutral character of the clonal mark. The sensitivity of clonal abundances to biases in SC loss and replacement provides a potential therapeutic screening strategy for further exploration in which using a simple histochemical technique to quantify the ratio of partial to fixed clones and the identification and quantification of stem cell expansion and crypt fission events could provide evidence for early changes in colorectal crypt SC homeostasis prior to overt tumour initiation, particularly in those patients with germline *APC* mutations.

## Funding Sources

This study was funded by the Newcastle University Centre for Ageing and Vitality supported by the BBSRC, EPSRC, ESRC, and MRC as part of the cross-council Lifelong Health and Wellbeing Initiative (JCM, DMT, LCG: L016354/1), the BBSRC (CS, AZ, DS, TK: BB/008200/1), The Urology Foundation (AS), the Rosetrees Trust (AS), the Wellcome Trust (BDS: 110326/Z/15/Z), The Wellcome Trust Centre for Mitochondrial Research [G906919] (DMT, LCG) and UK NIHR Biomedical Research Centre in Age and Age Related Diseases award to the Newcastle upon Tyne Hospitals NHS Foundation (DMT: IS-BRC-1215-20001).

## Conflicts of Interest

The authors confirm that there are no conflicts of interest.

## Author Contributions

C.S. performed the laboratory experiments. C.S., A.Z., A.S., E.A.S., and D.P·S designed the two-phase stochastic model, wrote the code and ran the simulations. J.C.M. collected the human colonoscopic biopsy material, D.M.T., T.B.L.K., and L.C.G. designed and directed the study, B.D.S. designed and performed the quantitative statistical analysis of the single-phase model, R.H., B.D.S., C.S., and L.C.G. interpreted the data and wrote the manuscript. All authors were involved in editing and approval of the final manuscript.

## Appendix A Supplementary data

Supplementary material Image 1
