# Supplementary material for: Predominant Asymmetrical Stem Cell Fate Outcome Limits the Rate of Niche Succession in Human Colonic Crypts
Source: eBioMedicine. 2018 Apr 25;31:166–73. doi: 10.1016/j.ebiom.2018.04.017 (PMC6013780; doi:10.1016/j.ebiom.2018.04.017)
Supplement: Supplementary file 1 — Supplementary material [file mmc1.docx]

**SUPPLEMENTARY INFORMATION**

1. Supplementary Theory Note 1
2. Supplementary Theory Note 2
3. Supplementary Figures
4. Supplementary References

**Supplementary Theory Note 1**

**Simplified model of drift dynamics of OXPHOS deficient clones**

To address the dynamics of OXPHOS-deficient clones in the human colorectal crypt, we make use of a model introduced by Lopez-Garcia et al.[^1^](#_ENREF_1) in which the effective stem cell (SC) compartment is characterized by a purely one-dimensional annulus of cells that line the circumference of the crypt base. In this model, SCs are lost and replaced by neighbors at a rate $\lambda$. The model is a simple caricature: In reality, *in vivo* live-imaging studies of the small intestine in mouse have shown that the niche is quasi one-dimensional with cells positioned near the border primed for differentiation while those at the base are poised for renewal [^2^](#_ENREF_2). However, under such conditions, the clonal dynamics quickly converges onto the effective one-dimensional dynamics with an “effective” SC number.

Applied to OXPHOS-deficient clones, it is important to take into account a potential proliferative advantage of SCs over their non-mutated neighbors. We therefore consider a refined model of the clonal dynamics introduced by Snippert et al. 2014[^3^](#_ENREF_3) in which there is a fractional bias, $\delta$, of mutated cells towards replacement and a corresponding bias of non-mutated cells towards loss. The theory is a straightforward extension of that developed by Lopez-Garcia et al. and the findings reproduced here. Details of the calculation can be found in the supplementary theory section of Snippert et al. 2014. Summarising the results of this study, for a non-neutral drift dynamics with a relative bias in the rate of SC loss/replacement, the probability of finding a partially labelled crypt with $0<n<N$ marked SCs at a time $t$ after the clonal labelling of a single SC is given by

$$P_{n}\left( t \right)=v^{n}\frac{2}{vN}\sum_{k=1}^{N-1} \sin\left[ \frac{\pi k}{N} \right]\sin\left[ \frac{\pi kn}{N} \right]e^{-\mu\lambda tf_{k}}$$

where $v=\sqrt{\frac{1+\delta}{1-\delta}}$, $\mu=\sqrt{1-\delta^{2}}$ and $f_{k}=2\left( \frac{1}{\mu}-1 \right)+4\sin^{2}\left[ \frac{\pi k}{2N} \right]$. Here $N$ denotes the effective SC number. The corresponding probabilities that the clone has become extinct (lost) or fixed (monoclonal) after a time $t$ is given, respectively, by

$$P_{0}\left( t \right)=\frac{2}{vN}\sum_{k=1}^{N-1} \frac{1}{f_{k}}\sin^{2}\left[ \frac{\pi k}{N} \right]\left( 1-e^{-\mu\lambda tf_{k}} \right)$$

$$P_{N}\left( t \right)=v^{n}\frac{2}{vN}\sum_{k=1}^{N-1} \frac{{(-1)}^{k+1}}{f_{k}}\sin^{2}\left[ \frac{\pi k}{N} \right]\left( 1-e^{-\mu\lambda tf_{k}} \right)$$

These results form the basis of our analysis of the clonal dynamics following mtDNA mutation.

To prepare for this analysis, we must first consider the internal dynamics of the mtDNA mutation accumulation itself. Notably, in contrast to a controlled genetic labelling system, there are several features that complicate the use of mtDNA mutation as a clonal marker. First, for mutations to be visible, they must reach a threshold of abundance. This imposes a time-delay between the initial acquisition of mutation and their detection and introduces an additional source of stochasticity into the dynamics. Second as a point mutation, it is reversible - the marker can disappear. Third, the acquisition rate of mutations may change substantially with age leading to changes in the effective induction frequency. Nevertheless, providing rates are low and timescales are separated, we can make some simplifying assumptions that can be checked by a more refined analysis (Supplementary Theory Note 2). First, the random partitioning of mtDNA between daughter cells will lead to a drift in relative mtDNA mutation number between generations with an average that increases linearly over time. This means that, to a first approximation, “induction” can be considered as a binary switch: mutant cells will not “hover” around the detection threshold. This is convenient as it circumvents the need to consider the internal dynamics of mtDNA accumulation within individual cells.

With this assumption, we then consider how the accumulation rate changes with age. In this case, we can focus first on the relative frequency of fully marked (OXPHOS-deficient) crypts. Although there is a large variation between, and even within, individual patient samples, overall there is a trend towards an exponential-like increase in OXPHOS-deficiency with age. This is emphasized by the corresponding increase in the number of partially labelled crypts, which provides a better indication of recent mutational activity.

Therefore, in the following, let us define $R\left( t \right)dt$ as the probability that a crypt SC acquires a new mutation between time $t$ and $t+dt$. Here and henceforth, we drop the denotation “effective” SC, although it is implicit. With probability $1/N$ such a clonally labelled SC will give rise to a clone that will drift to monoclonality over some typical time scale $T$. Therefore, neglecting the reversion rate of mutant SCs/crypts, which we take to be comparitively small, the frequency of fixed mutant crypts at time $t$ will be given by

$$F\approx\frac{1}{N}\int_{0}^{t} dtR(t-T)$$

Empirically, the data suggest an exponential increase in this frequency. We therefore introduce the parameterization $R\left( t \right)=R_{0}e^{\eta t}$, where $\eta$ denotes the effective mutation rate, i.e. the rate at which mutant cells rise above the detection threshold. As a result, it follows that

$$F=\frac{R_{0}}{\eta N}e^{\eta(t-T)}$$

From a fit to the data, we find that $\eta\approx0.052$ per year and $\frac{R_{0}}{\eta N}e^{-\eta T}\approx0.13$. In particular, if $\eta T\ll1$, it follows that $\frac{R_{0}}{N}\approx7\times{10}^{-3}$ per year.

Using these estimates, let us now turn to consider a more refined analysis using the predicted clonal dynamics of mutant SCs. Based on the inferred rate of mtDNA mutation, the probability of finding a fixed crypt in a patient of age $t$ would then be given by

$$Q_{N}\left( t \right)\approx\int_{0}^{t} dt^{'}R\left( t^{'} \right)P_{N}\left( t-t^{'} \right)=\frac{R_{0}e^{\eta t}}{\eta}v^{N}\frac{2}{vN}\sum_{k=1}^{N-1} {(-1)}^{k+1}\sin^{2}\left[ \frac{\pi k}{N} \right]\frac{\mu\lambda}{(\mu\lambda f_{k}+\eta)}$$

The corresponding distribution of partial crypts is given by

$$Q_{n}\left( t \right)\approx\int_{0}^{t} dt^{'}R\left( t^{'} \right)P_{n}\left( t-t^{'} \right)=\frac{R_{0}e^{\eta t}}{\eta}v^{n}\frac{2}{vN}\sum_{k=1}^{N-1} \sin\left[ \frac{\pi k}{N} \right]\sin\left[ \frac{\pi kn}{N} \right]\frac{\eta}{(\mu\lambda f_{k}+\eta)}$$

with the total number of fractional crypts given by $Q_{\mathrm{total}}\left( t \right)=\sum_{n=1}^{N-1} Q_{n}\left( t \right)$.

To apply these results, we now need to determine the effective SC number. As detailed in the main text, the distribution of partially marked crypts provides an indication of $N$. In particular, the position of the shoulder of marked crypts at a fraction of between 0.15 and 0.2 suggests an effective SC number of around 5-7. Here we take $N=5$ noting that the qualitative conclusions are largely insensitive of the precise value.

First, as a consistency check on the earlier fit, we can now use the inferred mutation rates to study the frequency of fixed and partial crypts, varying the SC replacement rate $\lambda$ to achieve an approximate fit. Previously, we took a defined fixation time, $T$. But, in practice, the theory predicts that fixation is not defined by a characteristic time scale, but follows a power-law dependence itself dependent on $\lambda$. Second, the theory above makes no prediction about the relative abundance of partial crypts. In principle, this fraction can vary widely depending on the effective SC number. If the SC number would be very small, the frequency of partially labelled crypts would be proportionately small. Vice verse, if the SC number would be very high, we would expect to capture a large number of partially labelled crypts at any given time. Taking the fits above for $\eta$ and $R_{0}$, the predictions of the frequency of partial and totals are shown in Supplementary Fig. S2A,B with $\lambda=0.6$ per year, taking the dynamics to be neutral and with a bias of $\delta=0.2$, respectively. Note that the coincidence of the two curves (partial and fixed) is highly unexpected and depends sensitively on the effective SC number, bias and $\lambda$, as shown. Small deviations of any of these parameters lead to a substantial departure of the curves, while the overall exponential dependence is preserved. Turning to the distribution of partially labelled crypts, the results are shown in Supplementary Fig. S2C,D for the same sets of parameters. Based on these findings, the clonal data would seem to be more consistent with neutral or near-neutral dynamics.

These results relied on several key assumptions; that the acquisition of visible OXPHOS-deficient cells could be modelled simply as a stochastic and irreversible event; that multiple induction events cannot occur in the same crypt; and that the output of the transit-amplifying cell compartment on the crypt wall provides a faithful read-out of the activity of the effective SCs at the crypt base. To challenge these assumptions, we turn to a multiscale stochastic model.

**Supplementary Theory Note 2**

**Multi-scale stochastic modelling of mtDNA clonal expansion and stem cell niche succession**

We developed a multi-scale stochastic model of mtDNA mutation clonal expansion by random genetic drift and SC niche succession within colonic crypts. The model was designed and run in MATLAB (version 7.14.0.739 MathWorks, Massachusetts, United States) and was based solely on experimentally derived parameters. The model source code is available from the authors upon request.

The model takes into account different scales of the human colon. The colonic tissue is comprised of individual colonic crypts, each containing approximately 2500 cells[^4-6^](#_ENREF_4). During the histochemical experiments it was established that OXPHOS-deficient cells are sometimes found together as clusters, however having established that these clusters have the same clonal origin [^7^](#_ENREF_7), they were counted as single crypts. This circumvented the need to include crypt fission into the model so we could model the crypts independently.

Out of the 2500 cells that constitute the crypt, we suppose that 5 function as effective SCs (determined in Fig. 2), which occupy the SC niche and are self-renewing, while the others are transit amplifying cells that give rise to the various differentiated cell types that are constantly lost from the crypt and replaced by SC division. We assume that only those mtDNA mutations that occur and expand in the SCs (but are also present in their progeny cells in the crypt) survive for long enough to allow clonal expansion to take place in order for OXPHOS deficiency to be observed. Therefore, only the SCs of the crypt are explicitly modelled.

At “time zero”, all copies of mtDNA within SCs are considered wild-type. SCs are known to have an mtDNA copy number at least 4-fold lower than fully differentiated cells [^8^](#_ENREF_8) with Lgr5 SCs having been shown to contain very few mitochondria [^9^](#_ENREF_9). We, therefore, set the mtDNA copy number at 200 per SC in this model. As the most likely source of mtDNA mutations are errors of the mtDNA polymerase, at each SC division there is a defined mtDNA mutation rate which, following a parameter scan, we estimated at around 1.0x10^-5^ at time zero, increasing exponentially up to 6.0x10^-5^ mutations per mtDNA replication at 80 years of age. This rate was comparable to previous reports [^5^](#_ENREF_5)^,^[^10-12^](#_ENREF_10), and reflected previously reported age-related increases in mtDNA point mutations [^13^](#_ENREF_13), thought to be due the relative infidelity of the mtDNA polymerase compared to the nuclear genome [^14^](#_ENREF_14) and the observed age-related reduction in quality control systems such as mitophagy [^15^](#_ENREF_15)^,^[^16^](#_ENREF_16). At each SC division we imposed a defined probability of either symmetric SC fate outcomes where either both effective SCs are lost from the niche or both are retained within the niche (Fig. 3), or asymmetric SC fate outcome where one SC is retained in the niche and one is lost from the niche. (Note that, although asymmetric fate outcome does not influence the width of the clone on the crypt, within the current framework, it does impact on the rate of mtDNA mutation.)

Based on proliferation kinetics measures, the SCs in the niche replicate with a cell division rate of once per week[^4^](#_ENREF_4). MtDNA is randomly segregated during the cell division process (Fig. 3A) and therefore the mtDNA mutated molecule may be retained and given the opportunity to replicate and propagate at the next SC division, or be lost from the SC niche. Over multiple SC division events, a mutated mtDNA molecule can reach the threshold for phenotypic expression of OXPHOS deficiency which was set at 75% in accordance with the previous reports [^6^](#_ENREF_6) (Fig 3B). This cell is then recorded as being OXPHOS deficient. The mutation may be either fixed or lost even after OXPHOS deficiency has occurred due to the stochastic nature of the model. Since the number of SCs in the niche is assumed to remain constant with age, a balance between SC expansion and loss is necessary: It is possible for the SC fate outcome to be invariantly asymmetrical, or invariantly symmetrical (providing that there is balance between divisions where both cells remain and divisions where both cells are lost), as is any combination of the two. In our model, we force the number of SCs in the niche to remain constant by always following one type of symmetric cell fate outcome with a neighbouring stem cell in the niche.

**Simulations**

In preliminary simulations we established that ca. 30,000 independent crypt simulations were necessary to reach convergence, and this was therefore this was the number of simulations used throughout the study.

In each simulation, the SCs in the niche undergo 4171 divisions, which was based on the division rate of 1/week results in 80 human years. Before each division for each SC, a mtDNA mutation is randomly acquired according to the defined probabilities above. To save computational time, if there are no mtDNA mutation present in the crypt, mtDNA replication, segregation and SC division are not explicitly simulated (since there is only one possible outcome, namely a niche without any mutations). When a mtDNA mutation occurs (and since this is a random process, it is not necessary for it to occur during the simulation of a single crypt), the simulation of mtDNA replication and segregation begins. Before each SC division, the mtDNA molecules first double in number and are then segregated randomly to the two daughter cells. MtDNA replication and segregation processes are described by transition matrices, the mathematical description of which can be found below. During SC division the fate of each SC in the niche is either asymmetrical or symmetrical according to the respective probabilities. If the fate outcome is asymmetrical, and the mutated mtDNA molecules are segregated into the cell which leaves the niche, they are lost from the simulation. If they are segregated into the SC which remains in the niche, they remain in the simulation. When the fate outcome is symmetrical and both cells are kept in the niche, one of the other SCs in the niche is removed to keep the number of SCs constant. When the outcome is symmetrical and both are lost from the niche, one of the other SCs divides and both cells remain in the niche to keep the numbers constant.

**Model fitting**

The model was fit to the experimental data with the probability of asymmetric fate outcome *p_a_* as a free parameter (with values 0-1), using a simple discrete gradient based method. Probability of symmetric outcome *p_s_ = 1 - p_a_*.

**Transition matrices**

In each division cycle there are *N_tot_* mtDNA molecules in the cell, out of which any number can carry a mutation. In the doubling step, *N* mtDNAs are chosen to replicate, however some of them can replicate more than once (the choice is a random N-combination with repetitions from a set of size *N_tot_*). The transition matrix gives the probability that *i* mtDNA molecules carrying the mutation will be picked *j* times, and the non-mutant carrying mtDNA molecules *N-j* times. Each element in the matrix (size (*N_tot_+1)*(N_tot_+1)*) is calculated by:

$$T_{1}\left( i,j \right)=\left\{ \begin{matrix} 1 & ; i,j=0 or i,j=N \\ & \\ \left( \frac{i}{N_{tot}} \right)^{j}\left( \frac{N_{tot}-i}{N_{tot}} \right)^{N-j}\left( \begin{matrix} N \\ j \end{matrix} \right) & ;otherwise \end{matrix} \right.$$

where *i ∈ [0,N_tot_]* is the number of mtDNA molecules carrying a mutation in the SCs before mtDNA replication and *j ∈ [0,N_tot_]* is the number of mtDNA molecules carrying the mutation gained in the process of replication.

After replication of mtDNA, the 2**N_tot_* mtDNA molecules are divided into two sets of same size. The transition matrix gives the probabilities that out of the *k* mtDNA molecules carrying the mutation in the mother SC, *m* will be chosen for one daughter cell. The transition matrix is of size *(2*N_tot_+1)*(N_tot_+1),* and each element can be calculated by:

$$T_{2}\left( k,m \right)=\left\{ \begin{matrix} \begin{matrix} 0 \\ \\ \begin{matrix} 1 \\ \\ \frac{(\frac{N_{tot}+N}{2}-1)!}{(N_{tot}+N)!}\prod_{x=0}^{m-1} (k-x)\prod_{y=0}^{\frac{N_{tot}+N}{2}-1-m} (N_{tot}+N-k-y)\left( \begin{matrix} (N_{tot}+N)/2 \\ m \end{matrix} \right) \end{matrix} \end{matrix} & \begin{matrix} ;\left( m>k \right) or (m<k-(N+N_{tot})/2 \\ \\ \begin{matrix} ;k,m=0 or k=400,m=200 \\ \\ ;otherwise \end{matrix} \end{matrix} \end{matrix} \right.$$

where *k ∈ [0,2*N_tot_]* is the number of mtDNA molecules carrying a mutation before division in the mother SC and *m ∈ [0,N_tot_]* is the number of mtDNA molecules carrying the mutation in one of the daughter cells after division.

**OXPHOS deficient SC distribution model**

The SC niche model simulates the emergence of OXPHOS-deficient SCs and their expansion and extinction over time. However, the biological data attained from human colonic tissue biopsies is a measure of OXPHOS deficiency percentage of individual crypts when viewed in transverse cross sections. Depending on where the tissue was cut will determine at which level the crypt is observed. As the majority of the crypt is made up of SC progeny and differentiated cells, it is highly likely that it is these cells that are observed as opposed to the SCs at the base of the crypt. Therefore, a method for determining how the number of OXPHOS-deficient SCs translates to the OXPHOS deficiency percentage higher up the crypt was required in order for the model data to be comparable with the biological data.

‘Functional’ SCs divide to produce SC progeny that rapidly divide and differentiate while they are being shuttled up the crypt due to expansion in cell numbers. This is analogous to a ‘conveyor belt’ of cells that are eventually shed off into the gut lumen. It is reasonable to assume that there is a scaling up of the number of cells that are clonally derived from a single ‘functional’ SC due to expansion in cell numbers. As it has been shown previously[^17^](#_ENREF_17), within partially OXPHOS-deficient human colonic crypts there is a ‘wiggle’ of OXPHOS deficiency from the base to the top of the crypt. This was explained by increased and decreased symmetrical fate outcome of OXPHOS-deficient SC clones, creating a ribbon imprint of OXPHOS deficiency on the crypt wall. However, as there will also be competition between the SC progeny (derived from asynchronous SC divisions) that are rapidly dividing, it is likely that this will also contribute to the ‘wiggle’ that is observed within human colonic crypts. With this rationale, for each number of OXPHOS-deficient SCs that are present within the SC niche, there must be an associated probability distribution for the likely OXPHOS deficiency percentage observed.

Therefore, a model was developed that assumes there is a doubling of the number of cells until a certain number of cells is surpassed (to replicate the cell expansion process), starting with the number of ‘functional’ SCs. For each of the ‘functional’ SCs that are OXPHOS-deficient, random replication is simulated until the number of cells at the next level is reached. This process is repeated for each cell expansion level. The number of OXPHOS-deficient cells at the top level is recorded (observation level). The simulation was run 100,000 times to determine a distribution for the number of OXPHOS-deficient cells there are at the observation level. The data is then transformed into a probability distribution and used to convert the biological data into the number of likely OXPHOS-deficient SCs it likely represents. This probability distribution can also be reversed to relate OXPHOS-deficient SC number to likely % OXPHOS deficiency.

A method of random replication of cells to reach the subsequent level of the crypt was used to simulate the competition between equally fit competing populations within the crypt. The iterative equation used to carry out this random replication of OXPHOS-deficient cells is described in below

$$p=\frac{m}{t+r-1}$$

The equation describes how the probability is calculated to determine how many OXPHOS-deficient SCs will occupy the successive level in order to relate number of SCs to number of potential transit amplifying cells that are OXPHOS-deficient at the observation level. *p* = probability of OXPHOS-deficient SC dividing to take up a place in the successive level, *m* = number of mutated SCs, *t* = total number of cells at current level and *r* = replication event number where r = 1: t-1. The probability is recalculated for each dividing cell until the total number of cells at the current level is reached. The process is started again for each subsequent level with *m* initially set to the value it was at the end of the previous level.

**Supplementary Figure 1: Simplified model of drift dynamics of OXPHOS deficient clones**

**A,B** Frequency of partial (orange) and fixed (blue) crypts as a function of age taking the inferred values of the mutation rate $\eta$ and rate constant $R_{0}$ defined in the supplementary text and $\lambda=0.6$ per year. Panel A shows the results of neutral dynamics $\delta=0$ and panel B shows the dynamics with a bias of of $\delta=0.2$. **C,D** Relative abundance of partially labelled crypts shown for neutral dynamics (panel C) and biased dynamics with $\delta=0.2$ (panel D) with parameters set as above. Note that, in both cases, the inflections are a consequence of the exponential increase in the acquisition rate of mtDNA mutations. If the rate were constant, the neutral dynamics would involve a linear decrease, while for non-neutral dynamics, the inflection would tilt downwards. The exponential increase in the mutation rate reverses this trend.

**
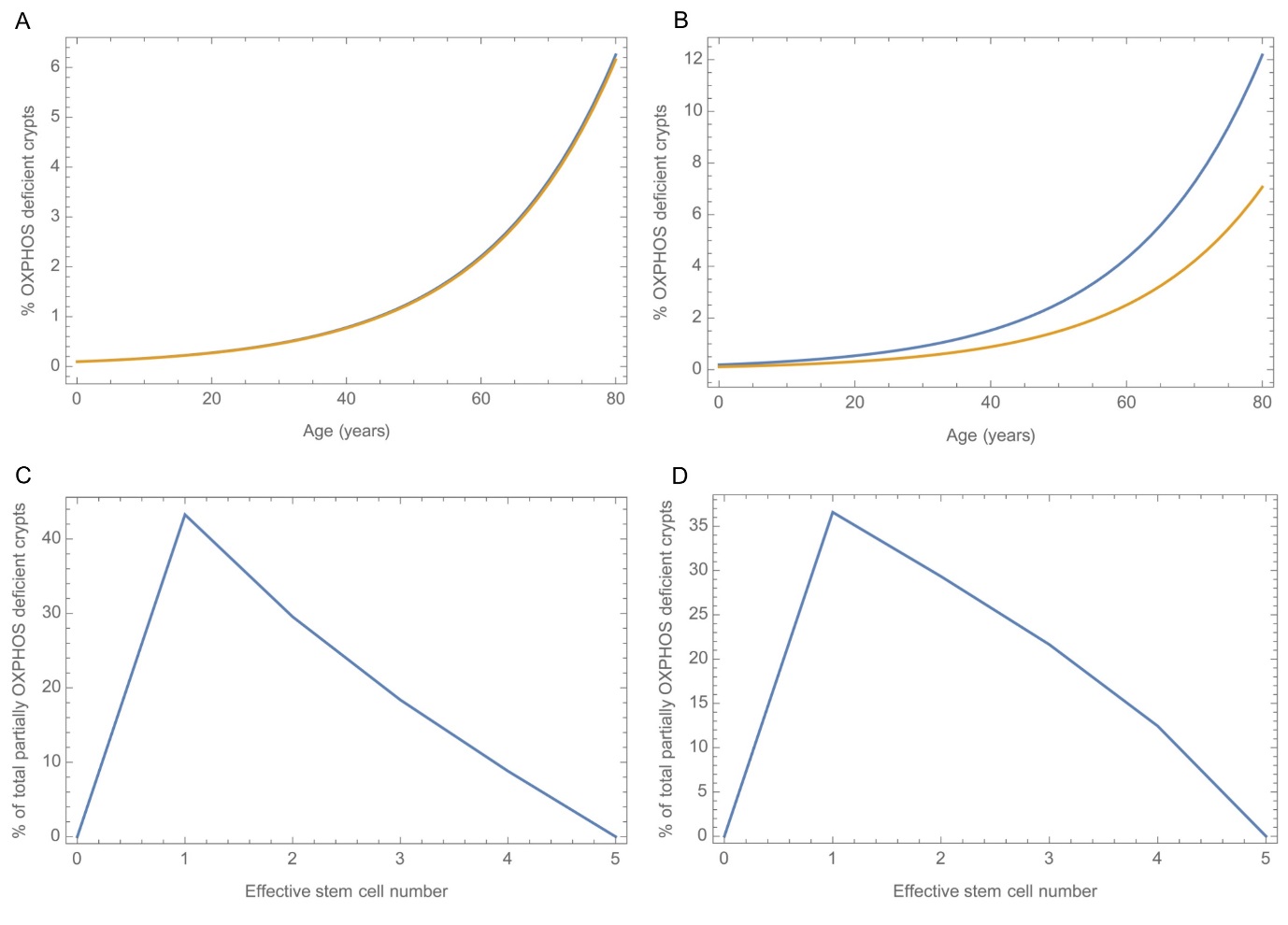
**

**Supplementary Figure 2: SC distribution model (related to Fig 3)**

(A) Model schematic which relates the number of OXPHOS-deficient SCs to the percentage OXPHOS deficiency observed within a transverse crypt within a sectioned biopsy. (Probability distribution for a crypt that contains (**B**) 3 SCs, (**C**) 4 SCs (**D**) 5 SCs (**E**) 6 SCs (**F**) 7 SCs, or (**G**) 8 SCs. (**H**) The distribution of partially OXPHOS-deficient within the models was fitted to the distribution as seen within the biological data using the SC distribution model. The method of least squares was used to determine the distribution that matched the biological data the best. (**I**) Least squares values for model fitting with 3-8 SCs. A model where 5 SCs were present within each crypt gave the best fit to the biological data.


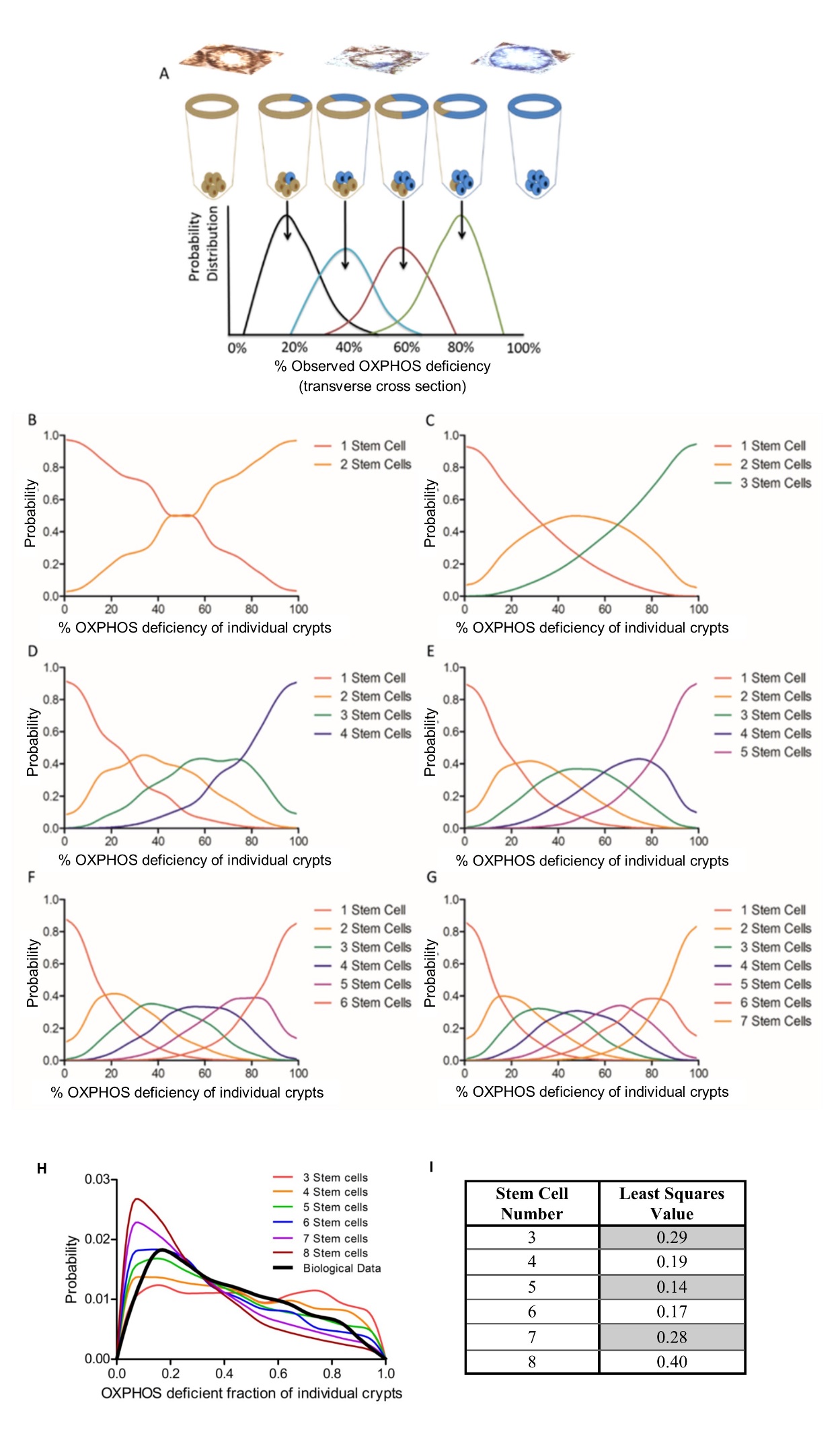


**Supplementary Figure 3: Optimised neutral drift model with 3-8 SCs (related to Fig 3)**

The neutral drift model was optimised for numbers of SCs ranging between 3-8 SCs per crypt. Best fit to biological data with (**A**) 3 SCs. (**B**) 4 SCs, (**C**) 5 SCs, (**D**) SCs, (**E**) 7 SCs, (**F**) 8 SCs.


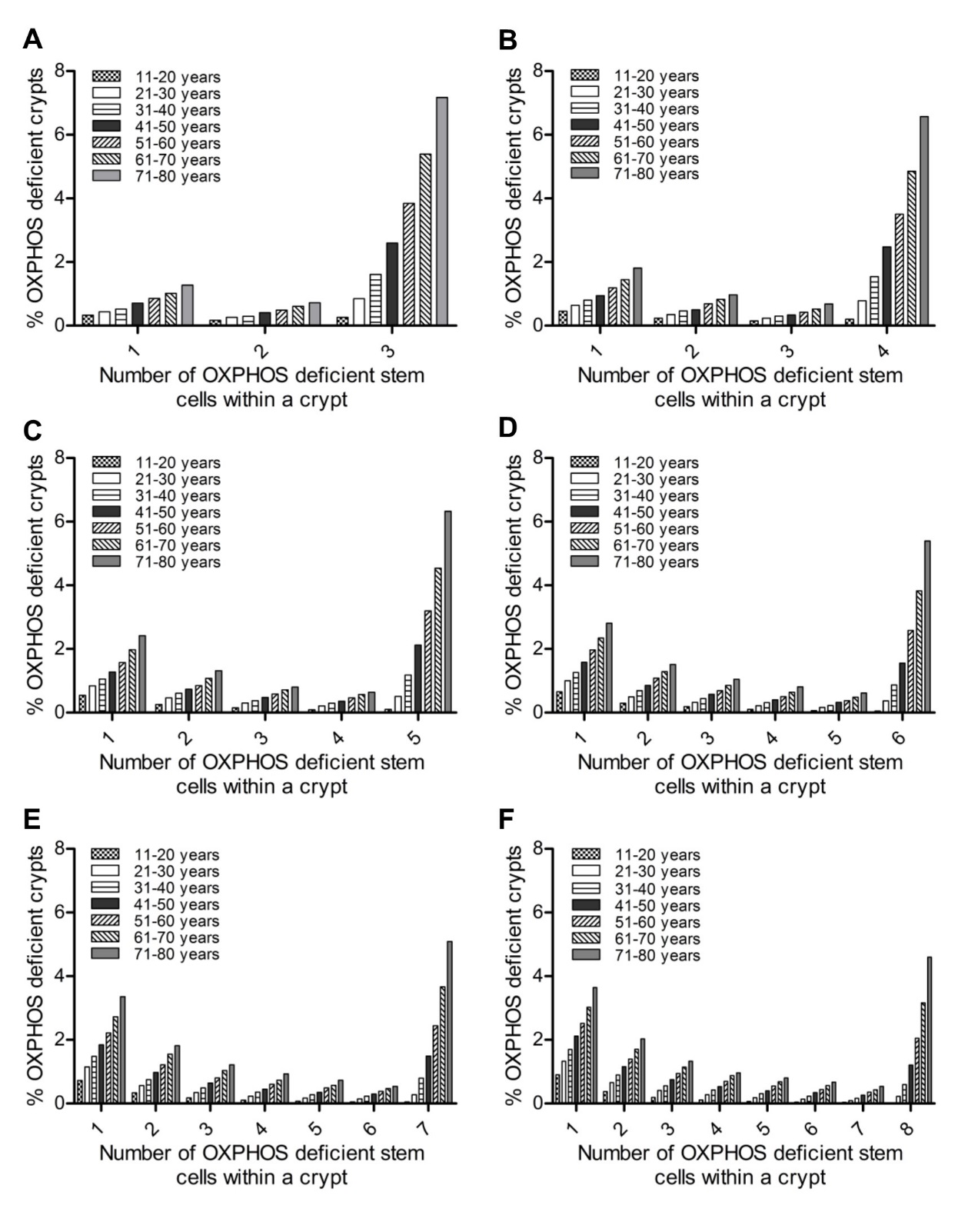


**SUPPLEMENTARY REFERENCES**

1. Lopez-Garcia, C., Klein, A.M., Simons, B.D. & Winton, D.J. Intestinal stem cell replacement follows a pattern of neutral drift. *Science* **330**, 822-825 (2010).

2. Ritsma, L.*, et al.* Intestinal crypt homeostasis revealed at single-stem-cell level by in vivo live imaging. *Nature* **507**, 362-365 (2014).

3. Snippert, H.J., Schepers, A.G., van Es, J.H., Simons, B.D. & Clevers, H. Biased competition between Lgr5 intestinal stem cells driven by oncogenic mutation induces clonal expansion. *EMBO Rep* **15**, 62-69 (2014).

4. Potten, C.S., Kellett, M., Roberts, S.A., Rew, D.A. & Wilson, G.D. Measurement of in vivo proliferation in human colorectal mucosa using bromodeoxyuridine. *Gut* **33**, 71-78 (1992).

5. Coller, H.A.*, et al.* High frequency of homoplasmic mitochondrial DNA mutations in human tumors can be explained without selection. *Nat Genet* **28**, 147-150. (2001).

6. Rossignol, R.*, et al.* Mitochondrial threshold effects. *Biochem J* **370**, 751-762 (2003).

7. Greaves, L.C.*, et al.* Mitochondrial DNA mutations are established in human colonic stem cells, and mutated clones expand by crypt fission. *Proc Natl Acad Sci U S A* **103**, 714-719 (2006).

8. Wanet, A.*, et al.* Mitochondrial remodeling in hepatic differentiation and dedifferentiation. *Int J Biochem Cell Biol* **54**, 174-185 (2014).

9. Barker, N.*, et al.* Identification of stem cells in small intestine and colon by marker gene Lgr5. *Nature* **449**, 1003-1007 (2007).

10. Coller, H.A., Bodyak, N.D. & Khrapko, K. Frequent intracellular clonal expansions of somatic mtDNA mutations: significance and mechanisms. *Ann N Y Acad Sci* **959**, 434-447 (2002).

11. Elson, J.L., Samuels, D.C., Turnbull, D.M. & Chinnery, P.F. Random intracellular drift explains the clonal expansion of mitochondrial DNA mutations with age. *Am J Hum Genet* **68**, 802-806 (2001).

12. Shenkar, R.*, et al.* The mutation rate of the human mtDNA deletion mtDNA4977. *Am J Hum Genet* **59**, 772-780 (1996).

13. Greaves, L.C.*, et al.* Clonal expansion of early to mid-life mitochondrial DNA point mutations drives mitochondrial dysfunction during human ageing. *PLoS Genet* **10**, e1004620 (2014).

14. Larsson, N.G. Somatic mitochondrial DNA mutations in mammalian aging. *Annu Rev Biochem* **79**, 683-706 (2010).

15. Quiros, P.M., Langer, T. & Lopez-Otin, C. New roles for mitochondrial proteases in health, ageing and disease. *Nat Rev Mol Cell Biol* **16**, 345-359 (2015).

16. Joseph, A.M.*, et al.* Dysregulation of mitochondrial quality control processes contribute to sarcopenia in a mouse model of premature aging. *PLoS One* **8**, e69327 (2013).

17. Baker, A.M.*, et al.* Quantification of crypt and stem cell evolution in the normal and neoplastic human colon. *Cell reports* **8**, 940-947 (2014).
